# Tumor Microenvironment on a Chip: The Progress and Future Perspective

**DOI:** 10.3390/bioengineering4030064

**Published:** 2017-07-21

**Authors:** Jungho Ahn, Yoshitaka J. Sei, Noo Li Jeon, YongTae Kim

**Affiliations:** 1 George W. Woodruff School of Mechanical Engineering, Georgia Institute of Technology, Atlanta, GA 30332, USA; ahnjungho0513@gmail.com (J.A.); yoshi.john@gmail.com (Y.J.S.); 2 School of Mechanical and Aerospace Engineering, Seoul National University, Seoul 151-744, Korea; njeon@snu.ac.kr; 3 Parker H. Petit Institute for Bioengineering and Bioscience, Georgia Institute of Technology, Atlanta, GA 30332, USA; 4 Institute for Electronics and Nanotechnology, Georgia Institute of Technology, Atlanta, GA 30332, USA; 5 Wallace H. Coulter Department of Biomedical Engineering, Georgia Institute of Technology, Atlanta, GA 30332, USA

**Keywords:** organ-on-a-chip, microfluidics, in vitro disease models, tumor microenvironment, drug screening, nanomedicine

## Abstract

Tumors develop in intricate microenvironments required for their sustained growth, invasion, and metastasis. The tumor microenvironment plays a critical role in the malignant or drug resistant nature of tumors, becoming a promising therapeutic target. Microengineered physiological systems capable of mimicking tumor environments are one emerging platform that allows for quantitative and reproducible characterization of tumor responses with pathophysiological relevance. This review highlights the recent advancements of engineered tumor microenvironment systems that enable the unprecedented mechanistic examination of cancer progression and metastasis. We discuss the progress and future perspective of these microengineered biomimetic approaches for anticancer drug prescreening applications.

## 1. Introduction

The cost of drug development has dramatically increased during the last several decades due to the inefficiency of current pre-clinical drug screening models [1]. Major disadvantages of conventional drug screening models are (i) the dissimilarity between two-dimensional (2D) in vitro cell culture systems and in vivo models; and (ii) the phylogenetic difference between human and animal models. Advanced 3D cell culture model systems have demonstrated advantages in providing more physiologically relevant conditions and more predictive ability [2,3,4]. The integration of microfluidic technology and cell biology research has recently reached a significant milestone with the development of “organ-on-a-chip” technologies that reconstitute organ-level in vivo characteristics [5,6]. Developing improved in vitro models through these innovative technologies will promote fundamental cancer research and accelerate drug discovery and clinical translation [7].

**Figure 1:**
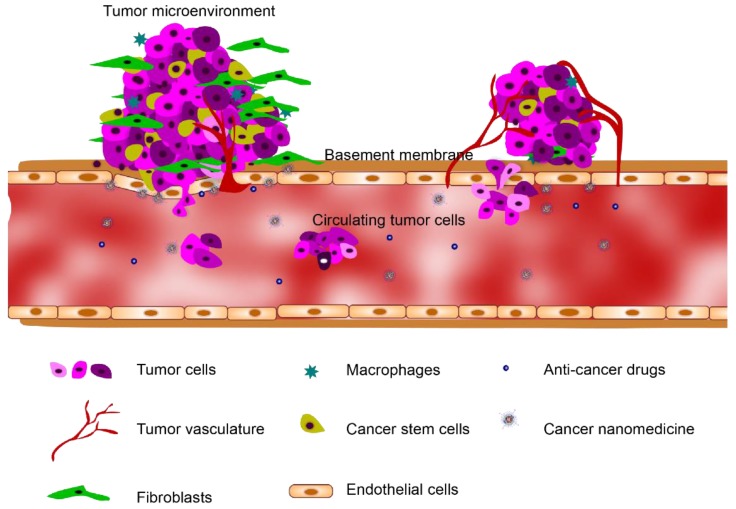
The tumor microenvironment (TME) heterogeneously consists of cellular and non-cellular components including the surrounding blood vessels, immune cells, fibroblasts, cancer stem cells and extracellular matrix (ECM).

A tumor microenvironment (TME) consists of a heterogeneous mix of cellular and non-cellular components including surrounding blood vessels, immune cells, fibroblasts, cancer stem cells and extracellular matrix (ECM) (Figure 1) [8]. The elucidation of the complex cellular interactions within the TME remains one of the main challenges in the treatment of cancer [9]. It has become increasingly recognized that the study of human cancer cannot be simplified to homogeneous collections of neoplastic cells, but must instead be studied as complex multicellular systems to properly reflect interactions between malignant and non-malignant cells [10,11,12]. This interplay between the tumor and the stroma has been recognized as a characteristic property of the TME, and this paradigm is now considered to be a hallmark of cancer biology [13,14,15]. Animal models are conventionally the gold standard for screening cancer therapeutics because of their capabilities to sustain the complex TME [16,17]. However, accurate mimicry of human tumorigenesis is extremely difficult, questioning the usefulness of existing in vivo models for therapeutic efficacy translation [14]. Meanwhile, recent advancements in the microengineering of TME using organ-on-a-chip technologies have enabled the development of pathophysiologically relevant human tumorigenesis models. In this review, we describe the most recent organ-on-a-chip approaches to study the tumor and its interactions with the surrounding microenvironments, including stromal cells, vasculature, and non-cellular components. We also highlight the applications of these leading-edge technologies to cancer drug/nanomedicine prescreening, and discuss the current challenges and future directions for these technologies.

## 2. Tumor Microenvironments on a Chip

Tumors interact with the surrounding microenvironments incessantly. Tumors typically consist of cancer cells and stromal cells (i.e., fibroblast and immune cells) that are nourished through vascular networking. Understanding the interactions between the tumor, stroma, and vasculature is key to the development of cancer treatments. In this section, we introduce microfluidic systems designed to mimic TME for studies of the interactions of tumor cells with stromal cells, endothelial cells, and non-cellular components.

### 2.1. Tumor-Stromal Interactions on a Chip

**Figure 2:**
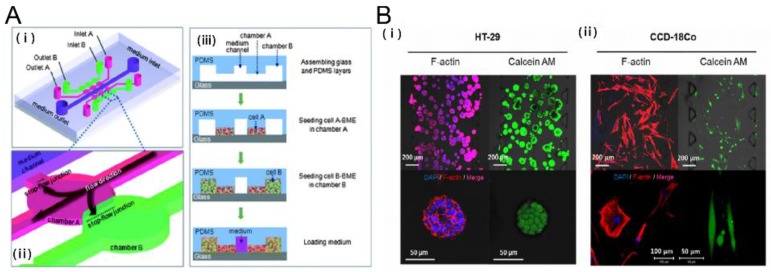
Tumor-stromal interactions on a chip. (**A**) 3D Microfluidic model to investigate the carcinoma associated fibroblast promoted tumor spheroid invasion. (i, ii) microfluidic chip design (iii) cell loading step. Salivary gland adenoid cystic carcinoma cell line (ACC-M) were co cultured with carcinoma associated fibroblasts (CAFs). ACC-M invaded CAF-embedded matrix in a spheroid fashion. However, ACC-M cells did not invade the adjacent matrix when co-cultured with the fibroblast cell line (HFL1) [21]; (**B**) 3D culture of tumor spheroids and fibroblasts in a compartmentalized microfluidic chip. (i, ii) Fluorescence images of HT-29 tumor spheroids and CCD-18Co human normal fibroblast cell line. HT-29 spheroids and CCD-18Co cells proliferated within the space of the corresponding channels over 5 days, during which their growth and interaction were monitored and characterized [26]. Reproduced with permission.

The stromal cells and tumor microenvironment modulate tumor sensitivity, which affects tumor cell signaling, proliferation, and drug resistance [18]. The non-malignant stromal cells include carcinoma associated fibroblasts (CAFs), pericytes, and adaptive immune cells [19]. Emerging microfabrication techniques enable the reconstitution of complex in vitro co-culture models for studying tumor-stromal interactions. Microfluidic systems provide greater spatial organization through controlled compartmentalization and higher sensitivity and control over the diffusion of soluble factors than traditional Transwell inserts [20]. Several key microfluidic tumor-stromal co-culture models have been developed to investigate the interactions. A microfluidic device designed to study salivary gland adenoid cystic carcinoma (ACC) cells and CAFs interactions when seeded in a 3D ECM has shown the potential of these platforms as a high-throughput parallel co-culture assay. This approach revealed that CAFs promoted ACC cell invasion into the 3D matrix, identifying a potential target for anti-cancer chemotherapies (Figure 2A) [21]. CAFs are considered to modulate tumor progression through cell to cell contacts and secretion of ECM components, growth factors and chemokines [19]. Therefore, understanding the interaction between CAFs and ACC cells will provide a potential target for anti-cancer chemotherapies. Another compartmentalized microfluidic chip was implemented to elucidate the cellular interactions between bone marrow stromal cells (HS5) and liver tumor cells (HuH7). It was observed that HS5 cells migrate towards HuH7 cells before the death of stromal cells upon contact with the tumor cells. It was found that the reactive oxygen species (ROS) level was significantly elevated in the co-culture system where the paracrine effect of the tumor cell-produced ROS caused the apoptosis of stromal cells [22]. Furthermore, it was revealed that mammary epithelial cells (MCF-DCIS) co-cultured with human mammary fibroblasts (HMFs) promote a transition from normal ductal carcinoma to an invasive one in situ; more importantly, only direct contact of HMFs with MCF-DCIS lead to the transition to invasion [23].

In addition to compartmentalized microfluidics, several other approaches have been developed to study cancer-stromal interactions. Continuous media supplementation allowed for 3D culture of a mixture of lung cancer cells and stromal cells for studies of the cancer-stromal cellular interactions [24]. A lung fibroblast paracrine loop was equipped with pneumatic microvalves to investigate the migration speeds of cancer cells, identifying the impact of transforming growth factor β1(TGF-β1) in the interaction between cancer cells and fibroblasts [25]. Recently, microfluidic systems offered a physiologically relevant in vitro tumor spheroid model to study the TME. Integrating 3D tumor spheroids with CAFs in proximity within a hydrogel scaffold exhibited mutual interactions (e.g., growth rate, ECM expression, morphological changes and increased migration in fibroblast) between the spheroids and fibroblasts (Figure 2B) [26]. In addition to fibroblasts, cancer cells actively recruit macrophages to remodel the TME and produce growth factors that increase the invasiveness of cancer cells [27,28]. MDA-MB-231 metastatic breast cancer cells and tumor-associated macrophages were embedded in collagen I and patterned within a microfluidic channel. It was found in this study that the tumor-associated macrophages invade the neighboring gels containing MDA-MB-231 cells, rather than migrating into the gels lacking cells [29].

As Stephen Paget suggested in the “Seed & Soil” hypothesis in 1889, tumor cells are like seeds being carried in all directions only if they settle into an appropriate soil. Stromal cells and ECM (soil) play a mutual supportive role in the initiation and progression of carcinogenesis (seeds). It remains extremely difficult to fully replicate the complex tumor-stromal interactions, although many microfluidic systems have created successful TMEs to study tumor-stromal interactions within microfluidic chips. Therefore, the critical elements to be mimicked or possibly ignored in a specific TME model should be carefully defined in a study to clarify the domain over which the study is relevant.

### 2.2. Tumor-Vasculature Interactions on a Chip

#### 2.2.1. Tumor Angiogenesis

**Figure 3:**
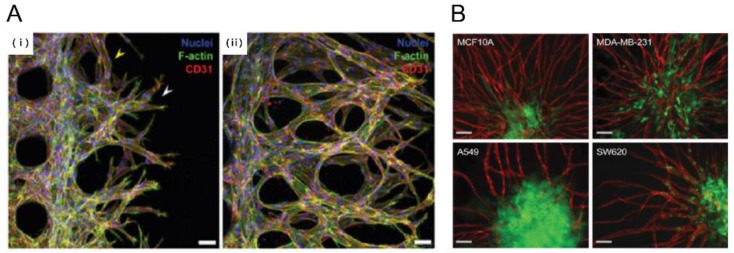
Tumor angiogenesis on a chip. (**A**) Human glioblastoma multiforme cells, (U87MG) were used to induce angiogenic sprouting. Fluorescence image shows angiogenic sprouts grown for 2 and 4 days under co-culture with U87MG cancer cells and human umbilical vein endothelial cells (HUVEC) (i, ii) [32]. Scale bar: 50 μm; (**B**) Pre-vascularized tumor (PVT) spheroid model. PVT spheroid model were introduced breast cancer (MCF10A, MDA-MB-231), Lung cancer (A549) and colon cancer (SW620). Representative fluorescence images of PVT spheroid model shows robust angiogenic sprouting. Various PVT spheroid showed different angiogneic sprouting behavior. Intravasation events were only observed for SW620 cancer cells [37]. Scale bar: 100 μm. Reproduced with permission.

Tumor growth and metastasis depend on angiogenic vascular networks, the growth of which are largely guided by chemical signals from tumor cells. Without the formation of new blood vessels, carcinomas neither grow well nor metastasize to colonial distant organs [30]. This rapidly growing angiogenic vasculature around the tumor is highly leaky, forming an aberrant vascular architecture [31]. Several 3D microfluidic systems have been developed to mimic these characteristics of cancer angiogenesis. Many of these microsystems allowed 2D endothelial monolayers to be vertically established in the side walls, which is designed to better image angiogenic sprouting into a 3D hydrogel. For example, a fibrin gel was either patterned into a microfluidic channel as a provisional matrix for endothelial sprouts or into a side channel for highly malignant human glioblastoma (U87MG). Endothelial cells (ECs) that were attached to the fibrin gel formed a pre-existing wall, 3D sprouting was promoted by the U87MG secretion factors. When compared to lung fibroblast-induced sprouts, U87MG-induced sprouts exhibited aberrant morphology, which is a general characteristic of cancer vasculature (Figure 3A) [32]. In addition, leukemic-cell-induced bone marrow angiogenesis has been demonstrated using a microfluidic chip, in which a collagen gel was filled into the middle channel, and U937, HL-60 and K562 cell lines were seeded into an upper channel to study their angiogenic induction. Upon forming the microenvironment of a bone marrow stromal cell line HS5, a unique morphogenic signature of angiogenesis was induced by different types of leukemic cells with or without co-culture with bone marrow stromal cells [33].

In addition to tumor secretion factors, shear stress is a critical factor to understanding tumor angiogenesis. One study investigated the combinatorial effect of shear stress and tumor-endothelial cross-talk on tumor angiogenesis in a 3D collagen hydrogel-based microfluidic platform, showing that decreased wall shear stress (WSS) increases both tumor-expressed angiogenic factors and endothelial permeability. More importantly, this shear stress-mediated tumor cell response was observed only in the presence of the endothelium [34]. In addition, early stages of tumor development can be accomplished with a glycosaminoglycan-based hydrogel culture system, which is capable of forming 3D tumor angiogenesis microenvironments. This system was implemented to reconstitute breast and prostate cancer vascularization, which showed tumor regressions comparable to those shown in vivo [35]. In addition, depletion of oxygen (hypoxia) in tumors due to excessive cell proliferation and dysfunctional vasculature promotes angiogenic signaling and tumor angiogenesis [30]. Oxygen status is therefore another critical determinant of tumor angiogenesis. Oxygen-controlled 3D alginate-based tumor models were developed to study angiogenic sprouting in hypoxic conditions, suggesting that pro-inflammatory pathways are a critical regulator of tumor and angiogenic response to hypoxia [36]. In order to study the complex multicellular interactions in a completely three-dimensional setting, a pre-vascularized tumor (PVT) spheroid model was introduced to investigate early events of solid tumor progression. PVT spheroids were formed through direct co-culture of EC and tumor cells and embedded in a fibrin gel mixed with human fibroblasts. After 7 days of culture, PVT spheroids exhibited robust sprouting angiogenesis (Figure 3B) [37]. Despite major advances in the development of tumor-angiogenesis-on-a-chip devices, the mechanisms by which tumor cells interact with the TME remain to be further studied.

#### 2.2.2. Tumor Transmigration through Endothelial Cell Lining: Metastasis

**Figure 4:**
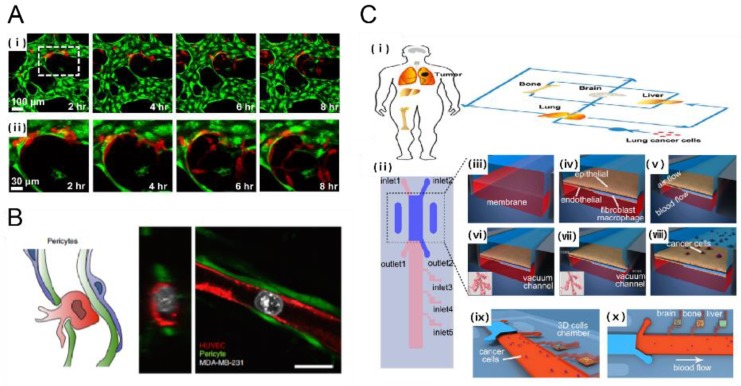
Metastasis on a chip. (**A**) A human 3D vascularized organotypic microfluidic system to study cancer cell extravasation (i) Cancer cell extravasation was monitored in real time within a vascular network (ii) magnified image [43]. Scale bar: 100 μm; (**B**) Human umbilical vein pericytes were cocultured with human umbilical vein endothelial cells to form pericyte-covered lumens. The extravasation rate from HUVEC-only cultures was significantly higher when compared to HUVEC-pericyte coculture [44]. Scale bar: 20 μm; (**C**) Design of biomimetic multi-organ chip (i, ii) multi-organ chip included an upstream “lung organ” and three downstream “distant organ” such as bone, brain, liver; (iii, iv) The microfluidic chip was compartmentalized using human epithelial and stromal cells cultured on separated side of a porous membrane in order to mimic (v–vii) physiological respiration in the microfluidic system; which was followed by the introduction of (viii–x) lung fibroblast cells to investigate lung cancer metastasis to distant organ [45]. Reproduced with permission.

In metastasis, cancer cells spread locally or distally by traveling through the blood or the lymphatic system to form a new tumor in other regions of the body [38]. This metastatic process involves a broad spectrum of invasion and migration mechanisms that include both single and collective cell migration strategies [39]. During metastasis, cancer cells disseminate to other parts of the body by entering the blood stream (intravasation) and getting out of the blood (extravasation) at proper metastatic sites [40]. Several microfluidic systems have been developed to mimic cancer cell transmigration through an endothelial cell lining. A 3D tumor vasculature interface was recreated in a microfluidic assay to characterize their interactions through tumor cell migration efficacy and endothelial permeability [41]. A microfluidic system was developed to mimic the specificity of human breast cancer metastasis into bone tissue by recreating a vascularized osteo-cell conditioned microenvironment with BM-hMSC that secreted a bone-like matrix [42]. This was further developed to study human metastatic breast cancer cell extravasation within a perfusable human microvascularized bone-mimicking microenvironment (Figure 4A) [43]. The method of implementing an in vitro model of metastasis in human microcirculation was given through multiple steps: early metastatic seeding, arresting, and transendothelial migration (Figure 4B) [44]. Recently, a multi-organ microfluidic platform was developed to reconstitute an in vivo microenvironment of lung cancer metastasis. This study successfully reproduced lung cancer growth, invasion, and metastasis to target distant organs including bone, brain and liver (Figure 4C) [45].

### 2.3. Tumor Interactions with Non-Cellular Components on a Chip

#### 2.3.1. Tumor-Extracellular Matrix Interaction

The extracellular matrix (ECM), the key non-cellular component of the TME, consists of several distinct components including proteins and glycoproteins [46]. Tumor growth is associated with mechanical alteration in the microenvironment, including increased matrix stiffness and aberrant interstitial fluid flow [47,48]. Various microfluidic models have incorporated 3D ECM matrix components and hydrogels into compartmentalized channels [49]. Fibrin gels [32,43], collagen gels [29,50], and matrigels [23,51] have been commonly used to reconstitute 3D microenvironments. These gels have the capacity to not only support tumor stroma such as fibroblasts and immune cells, but also to modify diffusion distance that allows for greater spatial control between different cell types. Furthermore, cancer cells in their intrinsic environment interact with a 3D ECM, characterized by physical parameters (e.g., porosity and stiffness) and by chemical parameters (e.g., adhesion site density and bound ligand concentration) [52]. Increased physical parameters of ECM alter the cellular force balance, leading to abnormal cell proliferation [53], and especially, increase in the rigidity of the matrix activates integrins and promotes Rho/ROCK pathway [54]. The crosstalk between the integrin/Rho pathway and Erk signaling cascade may induce self-sustaining process, leading to neoplastic disorganization of cancer tissue architecture [52,55]. In addition, it is crucial to note that our understanding of cell migration in 3D ECM is based largely on fibrous matrices, such as collagenous matrix found in the breast and other connective tissues [56]. However, other tissues are composed of non-collagenous, less structured materials [57]. For example, brain ECM is composed of hyaluronic acid and proteoglycans which form a more amorphous matrix [58]. Therefore, it is important to understand how their unique architecture contributes to tumor growth, and that the systematic consideration of 3D ECM properties should serve as an informative set of design criteria in the TME on a chip.

#### 2.3.2. Tumor-Chemokines Interaction

**Figure 5:**
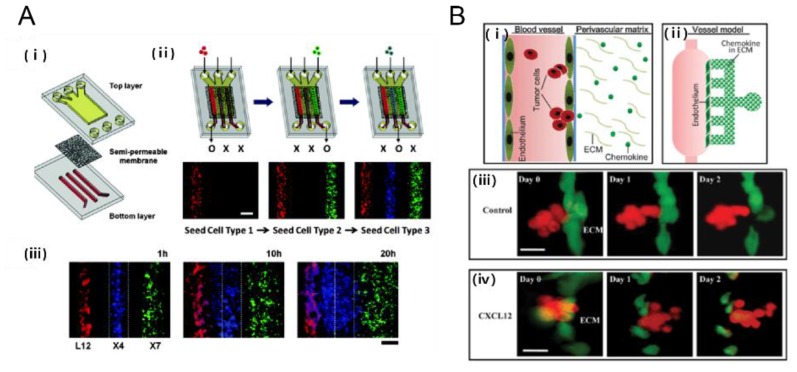
Tumor-chemokine interaction on a chip. (**A**) Chemotaxis in gradients induced cancer cell migration. (i) An in vitro model of tumor-stromal interaction engineerined in a microfluidic chip consisting of porous membrane; (ii) The cellular seeding procedure uses the following color-coded cells: Red (L12) CXCL12 producing cell, Blue(X4) CXCR4 expressing cells; and Green(X7) CXCR7 expressing cells (iii) Time lapse images show progressive migration of X4 cells toward L12 cells [60]. Scale bar: 200 μm; (**B**) A microfluidic device for study of transendothelial invasion of tumor aggregates by stimulation of chemokine CXCL12. (i, ii) Schematic representation of the device; (iii, iv) Transendothelial invasion of ACC-M aggregates induced by CXCL12. ACC-M aggregates could not transmigrate across HUVEC in the control but ACC-M aggregates transmigrated HUVEC and invaded into ECM when induced by CXCL12 [61]. Reproduced with permission.

Tumor cells rarely encounter a uniform environment. Instead, they often have to deal with a tangled chemical microenvironment where chemotaxis is guided by concentration gradients of chemokines and growth factors [59]. Previous conventional chambers used for gradient generation either lack sufficient resolution or are unable to maintain a steady gradient over time. Recent advancements in microfluidic systems allowed for the establishment of controlled chemical gradient profiles. Given that chemokine CXCL12 promotes CXCR4-dependent chemotaxis of cancer cells (MDA-MB-231), a compartmentalized microfluidic system that consists of two layers of PDMS channels separated by a semi-permeable membrane was developed to study CXCR4-dependent chemotaxis of cancer cells (MDA-MB-231) towards a CXCL12-producing source (Figure 5A) [60]. In addition, a microfluidic model that includes the endothelium and perivascular matrix containing CXCL12 chemokines was developed to observe the transendothelial invasion of tumor aggregates and analyze the extravasation process of salivary gland adenoid cystic carcinoma (ACC) cells exposed to CXCL12 (Figure 5B) [61]. Furthermore, lymph node metastasis serves as an indicator for distant metastasis in melanoma patients [62]. The receptor CCR7 and its ligand CCL21 have been implicated in the lymphatic spread of tumor [63] when CCR7 is expressed by melanoma cells [64] and when CCL21 is expressed by lymphatic endothelial cells [65,66]. Recently, the Boyden chamber incorporated into microfluidic systems were used to reconstitute the tumor-lymphatic microenvironment [67], in which luminal flow indirectly increases tumor cell (MDA-MB-231) transmigration by upregulating CCL21 expression. More studies underscore the role of chemokines in tumor migration [68]. Microfluidic technology is becoming increasingly more robust by creating more complex microenvironments with the superposition of multiple chemokines (e.g., CCR7-dependent chemotaxis which mediates metastasis) or growth factors [69,70,71].

## 3. Probing the Efficacy of Drug Delivery Using TME-on-a-Chip

3D microfluidic culture models are increasingly being used as prescreening tools for drug discovery including drug delivery and translation in oncology [72]. These models present more pathophysiologically relevant microenvironments of solid tumors in which the direct cell-cell interactions and metabolic mechanisms are better recapitulated in 3D multicellular spheroids than in conventional 2D cell culture models. We highlight the application of TME-on-a-chip models for cancer drug delivery and screening tools.

### 3.1. Microfluidic Platforms for Cancer Drug Delivery & Screening

**Figure 6:**
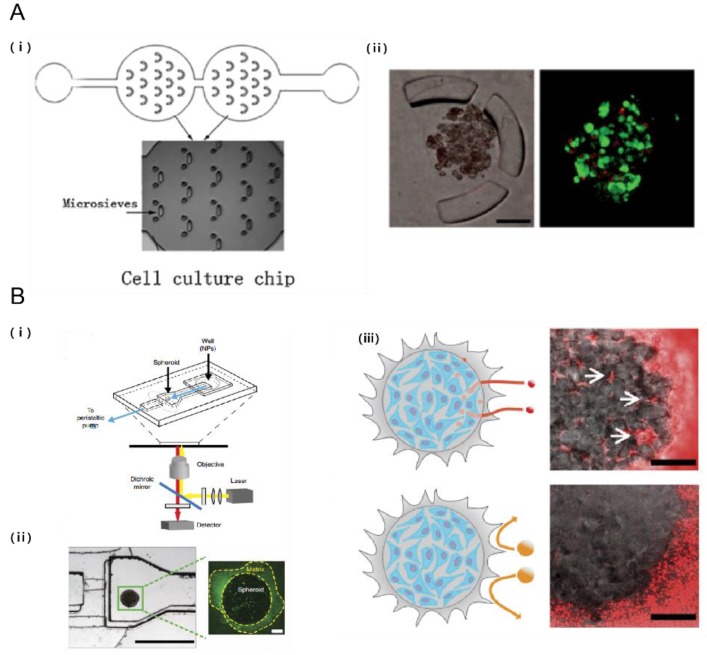
Probing the efficacy of drug delivery using TME on a chip. (**A**) Droplet-based microfluidic system for multicellular tumor spheroid formation and anticancer drug testing. (i) Schematic of the droplet formation and cell culture microfluidic chips. Each chamber contains 14 sieves for alginate droplet trapping; (ii) Breast tumor cells proliferating and forming multicellular spheroids while encapsulated in alginate beads. Tumor cells were perfused with doxorubicin and live/dead assay was assessed [75]. Scale bar: 100 μm; (**B**) Tumor on a chip provides an optical window into nanoparticle tissue transport. (i) Schematic of the microfluidic device; (ii) MDA-MB-435 breast cancer cell embedded within microfluidic device (iii) Effect of nanoparticle size on tissue accumulation. 40 nm fluorescent PEG-nanoparticles entered the tumor spheroid and accumulated in the interstitial spaces but 110 nm nanoparticles were excluded from the spheroid [93]. Scale bar: 100 μm. Reproduced with permission.

Conventional routes to screening compounds are a time-consuming and complex procedure [73]. Microfluidic systems have the potential to enable high-throughput drug screening in a controllable and scalable manner [74]. A droplet-based microfluidic system was used to form alginate beads with entrapped breast tumor cells. After gelation, the alginate beads were trapped in a microsieve structure for cell culture in a continuous perfusion system. This microfluidic system maintained a constant location for each bead to allow tumor cells to proliferate and form spheroids. The dose-dependent response of the tumor spheroids to doxorubicin, a common anthracycline, showed a higher survival rate in the multicellular spheroid culture compared to the conventional monolayer culture. (Figure 6A) [75]. Tumor spheroids have several structural, functional, and physiological similarities to tumors in vivo. 3D tumor spheroids enable them to communicate with each other as well as with their surroundings and provide an optimal environment for the cells to respond like they would in a tumor [76]. Furthermore, the inclusion of stromal cells to tumor cell culture showed a significantly higher drug resistance compared to when tumor cells were cultured alone [24]. Furthermore, pH and oxygen sensor integrated microfluidic systems allowed the monitoring of the long-term response of T98G human brain cancer cells to several drugs [77].

Aside from targeting the tumor cells directly, anti-angiogenic therapy, which suppresses tumor growth by disrupting nutrient and oxygen supply from the blood stream to the tumor, has widely been accepted as a potential treatment for cancer [78]. In particular, VEGF-induced and lung fibroblast-induced chemotactic responses of human umbilical vein endothelial cells (HUVECs) to different concentrations of bortezomib and other anti-angiogenesis drugs were tested in microfluidic systems [79,80]. To gain a better understanding of cancer drug screening, more physiologically relevant models need to be developed to reconstitute the complex interactions within the TME that is known to increase drug resistance. The majority of the microfluidic systems employed in drug delivery rely on diffusive drug release over an extended time period, as the drug administration period lasts from several hours to weeks. To better mimic a physiological drug release profile, various flow patterns need to be incorporated with multiple cell types that exist in the TME, as pulsatile flow patterns are prevalent in vivo.

### 3.2. Microfluidic Systems for Cancer Nanomedicine

Nanomedicine is the medical application of nanotechnology for delivering theranostic cargos to target tissues with increased drug stability and reduced systemic toxicity. Loading drugs into proper nanocarriers enhances the in vivo stability [81] and allows for the controlled release of drugs [82]. The preferential accumulation of nanoparticles in tumors is largely known to be due to the leaky tumor vessels and impaired lymphatic drainage via the enhanced permeability and retention (EPR) effect [83,84,85]. However, the heterogeneity of the TME affects the efficacy of passive targeting in drug delivery [86].

Recent approaches using active targeting were designed to address the challenges of passive targeting through utilization of ligand modifications to the surface of nanoparticles for specific affinity-based uptake by targeted disease cells [83]. The main mechanism underlying active targeting is the recognition of the ligand by its target substrate. The representative examples for commonly-used ligands include anti-bodies, proteins, peptides, nucleic acids and small molecules such as vitamins [87]. Active targeting has been efficiently used to promote target cell internalization of nanoparticles. Anti-HER2 targeting monoclonal antibody (mAb) fragments on the surface of liposomes strongly increase the uptake of nanoparticles in HER2-expressing cancer cells [88]. Recently, several in vitro studies showed a single nanoparticle can target multiple surface receptors [89,90,91].

Recent studies consider the importance of the TME properties when evaluating nanoparticles targeting tumor cells. A tumor-microenvironment on a chip (T-MOC) was used to investigate nanoparticle transport and the resulting variation to delivery efficacy due to changes in the TME properties including cut-off pore size, interstitial fluidic pressure, and tumor tissue microstructure [92]. To recapitulate the complex transport process around a tumor, the T-MOC employed a 3D structure formed by stacking microchannels with a porous membrane inserted. The capillary endothelium was cultured on the top layer, and the tumor interstitium and lymphatics were created in the bottom layer. Furthermore, incorporation of tumor-like spheroids into a microfluidic channel allowed for the real-time analysis of nanoparticle accumulation in pathophysiological flow conditions, showing that the penetration of nanoparticles into the tissue is limited by their diameter and that the retention could be improved by receptor targeting (Figure 6B) [93]. Notably, recent development in the synthesis of nanoparticles involves the discipline of microfluidics, enabling large-scale production of multicomponent nanoparticles with high reproducibility and size uniformity [94,95,96].

## 4. Microfluidic System Design Considerations for In Vitro TME Model

To reconstitute a physiologically relevant tumor microenvironment on a chip, several key parameters need to be taken into account. In this section, we will discuss unique characteristics of an in vivo TME to consider improvements to in vitro TME microfluidic models for drug delivery studies.

### 4.1. EPR Effect

The preferential accumulation of nanoparticles in a tumor is generally attributed to defective and leaky tumor vasculature [97] and dysfunctional lymphatic vessels in the tissue that cause poor drainage [98,99]. All of these factors are mainly known to contribute to the EPR effect, facilitating nanoparticle delivery to a solid tumor site [83,84,85]. Conventional in vitro 2D models however were unable to evaluate the full extent of the EPR effect.

### 4.2. Solid Tumor Stress

It has long been known that tissue stiffness is higher than normal in fibrotic solid tumors [100]. Tumors containing abnormally high amounts of collagen and other scaffolding proteins have been linked to several hallmark characteristics of cancer, including tumor growth, invasiveness, and metastasis [101,102]. Accumulation of mechanical stresses within the TME may lead to the constriction of intratumoral blood vessels, drastically reducing oxygen supply and increasing the risk of hypoxia and necrosis [103,104,105]. Moreover, vessel compression decreases blood flow, which also reduces the delivery efficacy of drugs to ultimately compromise therapeutic outcomes [106]. Apart from compression of intratumoral vessels, the solid stress directly affects cancer cells by increasing their apoptotic rate and reducing proliferation [105,107,108]. With the widely-known contribution of mechanical stress to tumor progression, biomechanical models of tumor growth have been developed to consider the generation and accumulation of mechanical stresses in tumors [109,110,111]. However, few microfluidic model-based studies focused on the incorporation of solid tumor stress, which is key to the design of physiologically relevant in vitro tumor models.

### 4.3. Normalization of Tumor Blood Vessels

The physiological consequences of tumor vascular abnormalities include temporal and spatial heterogeneity in tumor blood flow and increased fluid pressure [112]. These abnormalities promote tumor progression and lead to reduction in the distribution of an anti-cancer drug. Therefore, one of the main purposes to include vascular normalization into microfluidic platforms is to examine the phenotypic transformation of abnormal vasculature into a phenotype that closely resembles functionally normal blood vessels by increasing coverage of pericytes and the basement membrane, eventually decreasing vessel permeability [113,114]. Tumor vascular normalization repairs not only abnormal morphology but also the function of tumor vasculature, by correcting angiogenic signaling pathways [115]. However, normalized vessels with reduced fenestration may also hinder EPR-effect based delivery of large nanoparticles to the tumor site. Therefore, exploring the appropriate tumor vessel normalization may be needed to improve and balance nanomedicine delivery to a tumor site [116]. Previous models to investigate dynamic changes during tumor vasculature normalization heavily rely on computational models and mouse models [116,117]. Microfluidic platforms may provide a better understanding of the molecular, cellular, and functional changes during dynamic tumor vessel normalization with physiological relevance.

## 5. Conclusions & Future Perspectives

We reviewed recent approaches using microfluidic chips to study tumor interactions with various components of its microenvironment (fibroblasts, immune cells, endothelial cells, and non-cellular components). To build these in vitro TME model systems is essential to acquire a deeper understanding of the cellular and molecular mechanisms by which the TME contributes to tumor growth and metastasis. Traditional 2D in vitro systems, Transwell culture, and spheroid formation models that are used to mimic TME have shown limited effectiveness in predicting the efficacy of many candidate drug compounds [49]. Microfluidic models enabled us to study tumor microenvironment in real-time in a precisely controlled manner (e.g., oxygen concentrations and cytokine concentrations) [118]. These models can address the key challenges of conventional platforms and enable more complex yet critical studies with multi-parametric interactions including cell-to-cell and cell-to-matrix interactions within the TME. While much progress has been made for understanding tumor behavior and its many interactions, the TME-on-a-chip platforms are still to be improved to overcome several barriers: many devices are still complex to fabricate; integrated genetic quantification (e.g., running qPCR) in these platforms is too difficult to implement; there remains ambiguity in the required complexity of the system to yield physiologically relevant data; short culture times relative to in vivo lead to questions of physiological relevance; and it is difficult to obtain highly reproducible results with patient-specific samples. Microfluidic technologies continue to be developed and advanced to pave the way to a new generation of powerful in vitro experimental assays, which, when combined with in vivo validation, can deepen our fundamental understanding of cancer biology and facilitate the discovery of anti-cancer drugs to combat tumor progression and metastasis.

Several animal models have been used to investigate the EPR effect [81], solid tumor stress [105] and normalization of tumor blood vessels [115]. However, few microfluidic models were developed incorporating all these factors. Recently, one representative model of TME-on-a-chip system that incorporates tumor-like spheroids into a microfluidic chip showed the penetration of nanoparticles into a tumor tissue with physiological flow conditions, validating the EPR effect in vitro [93]. Likewise, the next generation of microfluidic devices would possibly use patient derived cells and extracted non-cellular ECMs with the use of multiple biochemical, biophysical and biomechanical cues that are characterized in cancer (EPR effect, solid tumor stress, and blood vessel normalization). This approach will also be integrated with high detection efficiency and high throughput technologies to enhance the clinical relevance of microfluidic technologies for cancer detection.
